# Cheiloplasty

**Published:** 1868-03

**Authors:** 


Cheiloplasty.—Fanny S., æt. 11. Three years ago, during an attack of typhoid fever, mortification took place in the lower lip, the jaw-bone became necrosed, the teeth dropped out, and a large gap was made in the lower lip. Such a result as this may occur in the low depressed state of the system attendant upon typhoid fever, protracted dysentery, scarlet fever, and other disorders producing a great drain upon the general vitality, in consequence either of the suffering occasioned by the disease, or from the want of nourishment. Under such circumstances mortification takes place very frequently on the lip, in the upper or lower jaw, or both; sometimes in an extremity, in the top of the nose, or in an ear. In some cases such effects are brought about by ptyalism. From its exceeding liability to set up inflammation apt to terminate in this way, mercury is dangerous when exhibited in low conditions of the system.

In remedying this deformity it will be necessary to dissect up the integuments from the lower jaw for some considerable distance, so as to bring the contiguous surfaces of the gap in apposition with each other. Unfortunately, the tissue around the opening is nodular, and consequently is exceedingly liable to slough, which is the great danger in cases of this kind.

The child was placed under the influence of chloroform, and the gap closed by sliding flaps forward, the parts being liberated thoroughly from the jaw-bone. The central wound was closed by three interrupted and three twisted sutures, the two lateral wounds being gently closed by the interrupted suture. The chief risk, after the operation, will be from erysipelas. She has been on the use of the tincture of the chloride of iron as a prophylactic.

